# Understudied Health Risks of High-Impact Pollution Weather: The Case for Revising Short-Term PM_2.5_ Standard and Indexes

**DOI:** 10.34133/hds.0460

**Published:** 2026-08-25

**Authors:** Pengfei Li, Jingyi Wu, Yongkang Yang, Tao Xue

**Affiliations:** ^1^ National Institute of Health Data Science, Peking University, Beijing 100191, China.; ^2^ Advanced Institute of Information Technology, Peking University, Hangzhou 311215, China.; ^3^ PKU-WUHAN Institute for Artificial Intelligence, Wuhan 430074, China.; ^4^ Institute for Artificial Intelligence, Peking University, Beijing 100871, China.; ^5^ Institute of Reproductive and Child Health, National Health Commission Key Laboratory of Reproductive Health and Department of Epidemiology and Biostatistics, Ministry of Education Key Laboratory of Epidemiology of Major Diseases (PKU), School of Public Health, Peking University Health Science Centre, Beijing 100191, China.; ^6^ State Environmental Protection Key Laboratory of Atmospheric Exposure and Health Risk Management, Centre for Environment and Health, Peking University, Beijing 100871, China.

## Abstract

In the Anthropocene, high-impact weather (HIW) events are increasingly compressing massive air pollution loads into narrow temporal windows, posing acute health risks that conventional environmental health frameworks were not designed to capture. Despite growing recognition of these dangers, critical gaps persist in the nowcasting of atmospheric health risks, as existing tools such as the air quality index (AQI) and the global burden of disease (GBD) framework remain anchored in chronic exposure paradigms and long-term concentration averages. This manuscript focuses on a pressing standard-index paradox in China: While air quality guidelines/standards (AQG/AQS) are being tightened, the risk-communication AQIs have largely remained static, creating a widening gap in public health protection. As a pilot study, leveraging a recently developed global function between short-term PM_2.5_ and cardiovascular mortality (CVD), we conducted a trade-off analysis across AQI cutoff values and identified that the lower boundary for moderately polluted events yields the highest leveraging efficiency. Under the prevailing ambient AQS, the optimal warning threshold is approximately 90 μg/m³, suggesting that health-protective warnings should be extended to cover some slightly polluted days as well. These findings underscore that nowcasting the health risks of acute air pollution episodes is of substantial public health importance, and that a risk-based warning strategy should be considered to replace China’s current AQI-based communication framework.

## Introduction

In the Anthropocene, we are witnessing a fundamental shift in the global burden of diseases (GBDs). High-impact weather (HIW) events, including heatwaves, wildfires, and dust storm, are increasingly frequent. They have become the primary drivers of acute pollutant spikes [e.g., wildfire/dust fine particles (PM_2.5_) and warm-season ozone episodes] that challenge the limits of human physiological resilience. Traditionally, environmental health policy has been focused on chronic exposure models, relying on long-term concentration averages. However, as climate change compresses massive toxicological loads into narrow temporal windows, there is a strategic imperative to pivot toward acute risk management.

The World Meteorological Organization (WMO), in collaboration with World Health Organization (WHO) and other agencies, has launched an initiative to establish an early warning system for health, which highlights meteorological variables including floods, typhoons, and temperature extremes [1]. Yet, a critical gap persists in the “nowcasting” of atmospheric constituents. This neglect leaves populations vulnerable to air pollution episodes that carry mortality risks comparable to major HIW events.

More and more assessments of air pollutants relied on the GBD framework [2,3], which, however, may inadvertently mask these acute dangers. By relying on annual average concentrations to estimate risk, the GBD “averages out” the destructive potential of short-term extremes. Furthermore, traditional environmental management tools like the air quality index (AQI) and short-term air quality guideline/standard (AQG/AQS) may not be health-oriented. For instance, WHO’s short-term AQGs are indirectly derived from the long-term ones, which, in contrast, are based on health effects of low-concentration exposure [4].

AQI is a widely used publication communication tool to nowcast how dangerous the air pollution is for human daily life. To make it more health-oriented, a few cities (e.g., Hong Kong) have developed the air quality health index (AQHI), which are mostly derived from a local epidemiological investigation by a multi-pollutant linear model on acute exposures [5]. However, AQHI suffers from technical limitations including the lack of generalizability (i.e., only data-rich cities can develop their own AQHI) and the out-of-distribution problem for rare HIW events (i.e., AQHI is statistically derived from the majority of atmospheric conditions but applies to the rare minority). As a result, the ambient AQS (AAQS)-AQI system should be improved to provide more effective protection against acute exposure to air pollutants during HIW events.

## Technical Barriers

Toward accurately nowcasting health risk of air pollution during HIW events, multiple technical barriers are addressed in environmental health science. Those barriers are rooted in complexity of acute exposure during HIW events. The relevant risk assessment should consider the nonlinearity, collinearity, and lag-distributed effect, which are briefly as follows.

• Nonlinearity: More and more evidence show that the health effects of air pollution are nonlinear, which is a key barrier toward the generalizability of the commonly reported linear estimates. Some policy-relevant nonlinear features are steep low-concentration effect, saturation effect, and tipping-point effect. A good example is the recently developed global function between short-term PM_2.5_ and cardiovascular mortality (CVD) [6]. First, the marginal health risk is steepest at the lower end of the concentration spectrum, even below the current WHO AAQS. Second, at moderate to high concentrations, the risk curve exhibits saturation of biological molecular pathways. Third, after temporarily flattening, the marginal risk sharply rises again at extreme levels. Ignoring nonlinearity, global linear fitting fundamentally underestimates hazards in relatively clean environments and miscalculates the danger of acute peaks.
• Collinearity: In HIW scenarios, meteorological factors and pollutants are highly coupled. The physical or chemical processes lead to compound exposure to multiple HIW events, such as heatwave–ozone, drought–wildfire, cold–dust, and ozone–precursors (e.g., nitrogen oxides and volatile organic compounds) pairs [7]. The collinearities make it difficult for single-pollutant models to isolate risk and guide specific interventions.
• Lag-distributed effect: There is a well-documented physiological delay between acute exposure and response. What is more, in air pollution epidemiology, “death displacement” or “harvesting” means that short-term spikes act as triggers for underlying vulnerabilities, with mortality manifesting days or weeks after the event. Nowcast systems that ignore these distributed lag effects fail to capture the true burden of HIW episodes.

To explicitly demonstrate the potentially underestimated health risks arising from the AAQS-AQI paradox and to provide a quantitative framework for identifying more effective warning strategies, we conducted a pilot study evaluating ongoing revisions to China’s AAQS-AQI framework. Building upon the recently developed nonlinear PM_2.5_–cardiovascular mortality exposure–response curve and AQI optimization approach described in our previous study [6], we adapted the approach to the Chinese context and utilized a novel efficiency index to quantify the trade-offs between alert fatigue and avoidable health burden under different AAQS-AQI thresholds.

## A Pilot Study

**Fig. 1.**
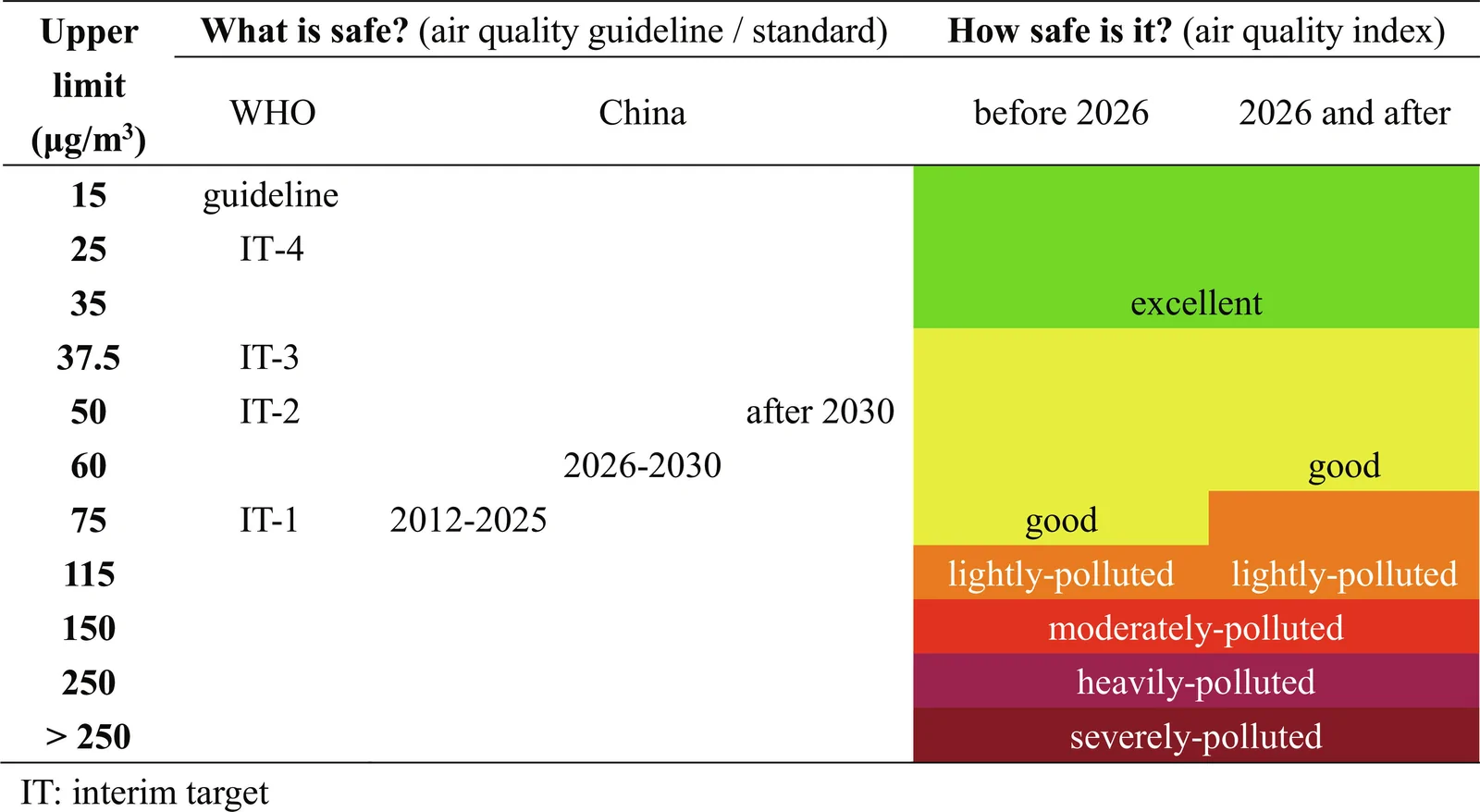
Air quality guideline/standard and air quality index on 24-h concentration of PM_2.5_, recommended by the World Health Organization (WHO) and China. IT, interim target.

China is currently evolving its national AAQSs to align more closely with the 2021 WHO AQGs, and a recent draft update proposes more stringent national air quality standards alongside corresponding revisions to the AQI based on the new short-term AAQSs [8–10]. This transition shows a typical standard-index paradox: While AAQSs are tightening, the risk-awareness AQIs mostly remain static, creating a gap in public protection. Using PM_2.5_ as an example, the paradox is shown in Fig. 1. By lowering the AAQS to 60 μg/m^3^, more days will be recognized as unsafe with the polluted-level AQIs, whereas the concentration threshold (>150 μg/m^3^) for warning of an event predicted as heavily polluted or above remains the same. As a result, after revising the AAQS-AQI system, the warned fraction out of all unsafe events will be reduced. This revision is advantageous to avoid alert fatigue, but disadvantageous due to the lack of protection. Facing the trade-off, whether the policy’s efficiency is improved or not needs to be evaluated by a quantitative health impact assessment.

**Fig. 2.**
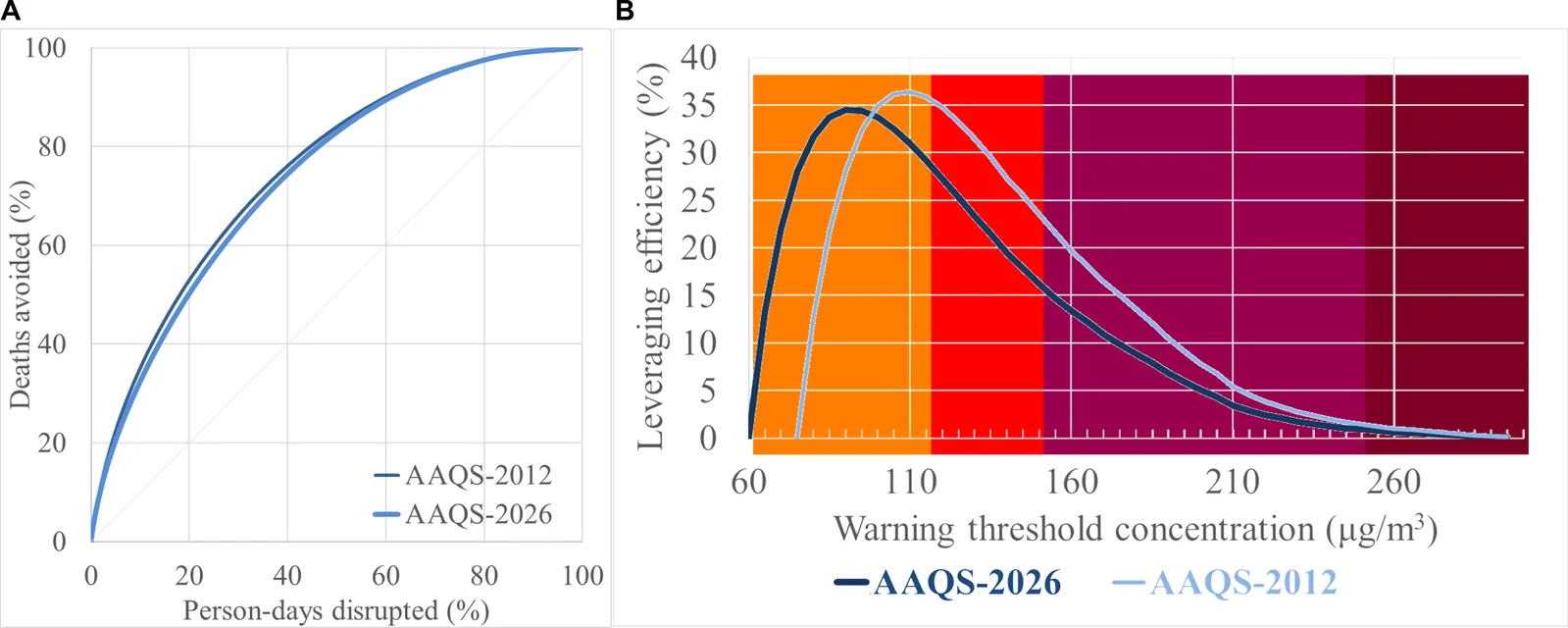
The trade-off analysis on different warning thresholds for the cardiovascular deaths attributable to short-term PM_2.5_ exposure in 2023. The numbers are derived from a previous study. (A) ROC-like trade-off curve between person-days disrupted and deaths avoided by a specific warning threshold concentration. (B) Their differences, which are defined as the leveraging efficiency, across various warning thresholds.

Based on the recently developed PM_2.5_–CVD curve, we calculated the national attributable burden in 2023 using the method as described in our previous study [6]. We conducted similar trade-off analysis: The social disruption was defined as the warned percentage out of all unsafe person-days, and the health benefit as the avoided percentage out of all CVD deaths attributable to PM_2.5_ exposures beyond the AAQS level (Fig. 2A). Daily PM_2.5_ exposure data, measured as 24-h average PM_2.5_ concentrations, were obtained from the MERRA2_CNN_HAQAST dataset. Population distribution data were derived from the high-resolution LandScan database, and cardiovascular mortality data were sourced from the GBD study. All datasets were spatially harmonized onto a unified grid covering China. Additional details on data sources and modeling procedures are provided in our previous study [6]. Specifically, the theoretical minimum risk exposure level (TMREL) was adapted to the Chinese context using AAQS-2012 (75 μg/m^3^) and AAQS-2026 (60 μg/m^3^).

Under the traditional AAQS, warnings for heavily or severally polluted events would be launched at 8.6% of unsafe person-days and avoid 32.1% of CVD deaths attributable to unsafe PM_2.5_ exposures. In contrast, the 2 percentages are reduced to 5.4% and 21.7% under the new AAQS. Conceptually drawing on net-benefit approaches used in decision curve analysis [11] and threshold optimization methods in receiver operating characteristic (ROC) analysis [12], the leveraging efficiency of a specific warning threshold was quantified as a difference of the 2 fractions under a commonly adopted equal-weighting assumption. This metric can be interpreted as a net population-level benefit metric, reflecting the balance between health gains and social disruption. However, the equal-weighting assumption may not fully capture trade-off preferences under different policy or societal priorities, and alternative weighting schemes could yield different optimal warning thresholds. Among all AQI cutoff values, lower boundary for the moderately polluted event shows the highest leveraging efficiency. For all AQI cutoffs, shifting the AAQS-AQI system consistently leads to an efficiency reduction, which is from 23.5% to 16.3% given the current 150 μg/m^3^ warning threshold. Furthermore, under the current AAQS, the optimal threshold is about 90 μg/m^3^, suggesting that the warning coverage should be extended to some slightly polluted days (Fig. 2B). The lower threshold observed in China, compared with the global setting examined in the previous study [6], likely reflects differences in exposure distributions, warning standards, and the underlying disease burden structure.

Notably, this pilot study was a preliminary analysis to provide simplified quantitative evidence of the potentially underestimated health risks arising from the AAQS-AQI paradox, and to act as a showcase for overcoming aforementioned barriers. A definitive optimized AQI threshold specifically for various health outcomes should be built on detailed, in-depth analysis, rather than the present study. As a proof-of-concept analysis, we did not fully incorporate several important methodological challenges discussed above, which should be addressed in future warning-model development. Similarly, we also omitted detailed discussions on uncertainty, sensitivity, and robustness of our findings in the present analysis paper. Our readers should realize that conclusions drawn from this pilot study are questionable. They should take our analytical methods and not the estimated numbers.

## Policy Implication

To protect public health in an era of climatic extremes, we must shift to risk-based health nowcast warnings. These systems should guide the proactive allocation of medical resources—such as intensive care unit (ICU) bed capacity and ambulance dispatch—during acute pollution shocks, specifically targeting interventions for sensitive groups when levels exceed established safety thresholds.

Broadly, the implementation requires 3 strategic pillars. First, we should develop a mature exposure-response model to capture the lag-distributed, nonlinear effects of multiple correlated air pollutants while further incorporating the heterogeneity in toxicity of event-specific PM_2.5_ compositions, population vulnerability, and adaptive capacity. Second, we should incorporate an advanced cost-benefit optimization. Crucially, early warning for air pollution episodes will provide greater benefits in populous, relatively less-polluted regions, refuting the notion that “cleaner” cities do not require stringent nowcasting. Our estimation of health benefits assumed that warnings automatically induce protective effects, which may not fully reflect real-world conditions. Future studies incorporating behavioral responses and compliance dynamics are needed to refine estimates of optimal warning thresholds and associated health benefits. Third, nowcast accuracy must be verified against real-world hospital admission rates and clinical outbreak data, moving beyond simple risk assessment.

To sum up, nowcasting air pollution shocks’ health risk is of public health importance. Our study highlights the limitations of current AAQS-AQI systems and provides illustrative evidence for the potentially underestimated health risks arising from the AAQS-AQI paradox, together with a simple framework for identifying more effective warning strategies. To enhance health protection, a risk-based warning strategy should be considered to replace China’s current AQI-based air quality communication.
